# Music Education at School: Too Little and Too Late? Evidence From a Longitudinal Study on Music Training in Preadolescents

**DOI:** 10.3389/fpsyg.2019.02704

**Published:** 2019-12-18

**Authors:** Desiré Carioti, Laura Danelli, Maria T. Guasti, Marcello Gallucci, Marco Perugini, Patrizia Steca, Natale Adolfo Stucchi, Angelo Maffezzoli, Maria Majno, Manuela Berlingeri, Eraldo Paulesu

**Affiliations:** ^1^Psychology Department, University of Milano-Bicocca, Milan, Italy; ^2^Department of Humanistic Studies, University of Urbino Carlo Bo, Urbino, Italy; ^3^Negri-Calasanzio Middle School, San Siro, Milan, Italy; ^4^SONG onlus – Sistema in Lombardia, Milan, Italy; ^5^Center of Developmental Neuropsychology, ASUR Marche, Pesaro, Italy; ^6^NeuroMi, Milan Center for Neuroscience, Milan, Italy; ^7^I.R.C.C.S. Galeazzi, Orthopedic Institute Milano, Milan, Italy

**Keywords:** intensive training, cognitive development, educational psychology, phonological skills, language, visuo-spatial skills

## Abstract

It is widely believed that intensive music training can boost cognitive and visuo-motor skills. However, this evidence is primarily based on retrospective studies; this makes it difficult to determine whether a cognitive advantage is caused by the intensive music training, or it is instead a factor influencing the choice of starting a music curriculum. To address these issues in a highly ecological setting, we tested longitudinally 128 students of a Middle School in Milan, at the beginning of the first class and, 1 year later, at the beginning of the second class. 72 students belonged to a Music curriculum (30 with previous music experience and 42 without) and 56 belonged to a Standard curriculum (44 with prior music experience and 12 without). Using a Principal Component Analysis, all the cognitive measures were grouped in four high-order factors, reflecting (a) General Cognitive Abilities, (b) Speed of Linguistic Elaboration, (c) Accuracy in Reading and Memory tests, and (d) Visuospatial and numerical skills. The longitudinal comparison of the four groups of students revealed that students from the Music curriculum had better performance in tests tackling General Cognitive Abilities, Visuospatial skills, and Accuracy in Reading and Memory tests. However, there were no significant curriculum-by-time interactions. Finally, the decision to have a musical experience before entering middle school was more likely to occur when the cultural background of the families was a high one. We conclude that a combination of family-related variables, early music experience, and pre-existent cognitive make-up is a likely explanation for the decision to enter a music curriculum at middle school.

## Introduction

Music training involves many neurocognitive systems, like audition, vision, motor control and their integration. Over the last 20 years, there has been a considerable increase of interest in the relationship between such training and the maturation of cognitive skills. Two main streams of studies have either focused on the comparison of adult musicians with non-musicians or on the effect of music learning on cognitive development in children.

### Studies in Adult Musicians

Many studies have shown that professional instrumental players and even amateur players outperform non-musicians in cognitive domains related to music and auditory skills (Schön et al., 2004; Kraus and Chandrasekaran, 2010; Strait et al., 2010; Pantev and Herholz, 2011) and language processing, both at the level of phonetics (Alexander et al., 2005; Marques et al., 2007; Jäncke, 2012; Kühnis et al., 2013; Elmer et al., 2017) and prosody (Thompson et al., 2004; Lima and Castro, 2011; Park et al., 2015). This occurs also for other cognitive skills that one would not readily associate with music training: for example, verbal memory (Chan et al., 1998; Franklin et al., 2008; George and Coch, 2011; Talamini et al., 2016), visuo-spatial skills (Brochard et al., 2004), visual mental imagery (Aleman et al., 2000), and visual memory (Jakobson et al., 2008).

Besides the clear evidence related to auditory processes (Schön et al., 2004; Schellenberg and Moreno, 2010; Habibi et al., 2016), one of the most recurrent results for non-musical cognitive skills is the one of verbal abilities and, particularly, verbal working memory (Franklin et al., 2008; Jakobson et al., 2008; Tierney et al., 2008; George and Coch, 2011): indeed, musicians achieve a superior performance in tasks where the subvocal rehearsal component of working memory is important (Franklin et al., 2008; Talamini et al., 2016). This has also been shown by Franklin et al. (2008) who found that the musicians’ advantage in a working memory task was lost specifically during articulatory suppression; this supports the idea that a more efficient subvocal rehearsal is the underlying factor for the outstanding memory performance observed in musicians.

There is some evidence that these behavioral patterns may be accompanied by specific anatomical brain findings (Schlaug et al., 1995; Münte et al., 2002; Schlaug, 2015): for example, professional musicians were found to have a larger anterior corpus callosum, whose size seems to vary in relation with the age at which the music training started, a left-lateralized asymmetry of the planum temporale^1^ and greater volume of Helsch’s gyrus, Broca’s area, the Superior Parietal lobule and the Cerebellum (see Schlaug et al., 1995; Schlaug, 2015).

Diffusion Tensor Imaging studies have also shown a higher level of diffusivity – hence of structural connectivity – in professional instrumental players in the internal capsule (Schmithorst and Wilke, 2002), in the corpus callosum and in the superior longitudinal fasciculus (Bengtsson et al., 2005), in the cortico-spinal tract (Imfeld et al., 2009) and in the anterior portion of the arcuate fasciculus (Halwani et al., 2011).

### Effect of Music Learning on Cognitive Development in Children

There is also a wealth of studies suggesting that music training may have a sizeable effect on cognitive maturation during childhood; it remains to be established at what stage of the development this might be so, whether music affects cognition in a broad sense or whether the effect is specific for cognitive skills that one may readily associate with music (e.g., auditory processing).

Schellenberg (2004) investigated whether music training has an impact on the IQ in a wide sample of children randomly assigned to a music training group, to an art training group or to the control group: music training had a boosting effect on the IQ, while training in arts was more effective on social behavior. Two further studies by Schellenberg (2006, 2011) confirmed an association between IQ and the duration of music training.

The IQ is a lumped measure of several functions and the observation of superior IQs in musically trained subjects does not demonstrate *per se* a generalized cognitive boosting effect. Other studies have tried to pinpoint the cognitive domains on which music training might have an effect on cognitive development. Not surprisingly, positive effects were found on cognitive abilities that have a close relationship with music, for example auditory processing (Trainor et al., 2003), phonological awareness (Moreno et al., 2011b; Francois et al., 2013) prosody (Thompson et al., 2004). It has to be noted that phonological and prosodic skills represent higher order auditory skills.

However, as for adults, other studies have found effects of music training on domains that are not specifically “musical” in any obvious sense, like learning skills and memory: children trained with music lessons have better performance in verbal memory tasks (Ho et al., 2003; Roden et al., 2012), verbal intelligence (Moreno et al., 2011a), language processing (see Patel, 2003, 2011) visuo-spatial skills (Rauscher et al., 1997), arithmetic (see Vaughn, 2000) and reading skills (Corrigall and Trainor, 2011; Tierney and Kraus, 2013; Slater et al., 2014). Accordingly, these developmental results seem to support, like the data from adults, a generalized “boosting effect hypothesis” of music on cognition.

Yet, a few issues remain open with this literature. For example, it is still possible that some of the effects that music seems to have on non-music-related skills may still be mediated by cognitive functions that it is not too hard to associate with music. One obvious example is the one of music and reading. According to a recent meta-analysis (Gordon et al., 2015), music training would positively affect reading skills via its effects on phonological skills^2^. A similar caveat is supported also by the results of musically-based treatments on children with learning difficulty and disabilities (Overy, 2003; Register et al., 2007; Cogo-Moreira et al., 2012; Flaugnacco et al., 2015) in which children with reading deficits showed a post-treatment improvement not only in reading tasks, but also in phonological tasks. The data on the visuospatial skills, and particularly on visual memory, are not clear either. As pointed out by Roden et al. (2012), the research performed in school contexts has provided non-conclusive or conflicting results: the visuospatial advantage reported by Gardiner et al. (1996), could be due to the fact that in that study children were trained both in music and visual arts making it impossible to distinguish whether any advantage was due to music training, to visual art training or to their combination. In the same vein, another study focused on music training at school (Rickard et al., 2010) reported an effect on verbal memory, even though this may not be a long-lasting one: on the other hand, the same study could not find a sizeable effects of music training on visual memory skills (Roden et al., 2012).

Longitudinal studies designed to document brain morphometry changes associated with music training (e.g., Hyde et al., 2009; Habibi et al., 2018) revealed signs of brain plasticity together with group specific changes in behavioral performance, yet only for domains strictly related to music training (e.g., audition, motor skills). A further evidence along these lines comes from brain morphometry studies that found a significantly larger corpus callosum, a marker of more efficient inter-hemispheric traffic, in people who started a music training before 7 years of age (see Schlaug, 2015 for a review).

To summarize, data from adult musicians and developmental studies, even though with some caveats, seem to point to a generalized boosting effect of music training on cognition, a result that may not be that surprising if one considers that music training involves so many neurocognitive functions that it would be quite unrealistic to expect an impact only on one or few cognitive domains.

However, as discussed below, all the considerations about the effects of music training, with the exception perhaps of those based on the few available longitudinal studies^3^, suffer of a major lingering limitation: the inability to distinguish causes and effects, to determine in a conclusive manner whether the cognitive advantage seen in musically-trained children or in adults is a genuine effect of the training, whether it is a specific one or whether it is a spurious effect due to the fact that a future musician may decide to join an educational program with intensive music training because of his predispositions. If the latter hypothesis were correct, it would be tempting to concur with Schellenberg and his statement that “music training is better suited for studying pre-existing differences in terms of brain and cognitive development rather than training specific plasticity” (Schellenberg, 2011, p. 297).

### Aim of the Study

As mentioned, one main limitation of previous literature is that the empirical observations made and the implications inferred were based primarily on retrospective or cross-sectional studies. Yet, the same issues could be better addressed and discussed using carefully designed longitudinal prospective studies (Schellenberg, 2004, 2006, 2011; Corrigall and Trainor, 2011; Moreno et al., 2011a; Tierney and Kraus, 2014) where one takes into account both the family’s cultural/socioeconomic status, the cognitive skills of the kids under examination and the school teaching content. One such approach may better discriminate the contribution of natural and nurture related factors (Sameroff, 2010) in this area of cognitive developmental psychology.

This is what we tried to achieve with the present study. In the light of these considerations, and with the aim of making a further step toward a better understanding on whether music may have a specific boosting effect on cognitive functions, we designed a longitudinal quasi-experimental^4^ study based on the assessment of cognitive development in pre-adolescents with and without previous music experience who attended either a music or a standard curriculum.

The decision to concentrate our efforts on pre-adolescents over their attendance to the middle school was motivated by pragmatic reasons: the time of the middle school is the only occasion when the Italian education system offers any programed instrumental music training, i.e., 2 h per week in canonical curricula or 5 h per week, including 2 h of music in ensemble, for the music curricula in the middle school where our study was based^5^.

Participants in the experimental group were about to start a music curriculum in middle school and were compared to their classmates who attended a standard curriculum. This comparison allowed us to keep the possible confounders under control and to isolate, as much as possible, the effect of more intensive music training. Sampling the children by their choice to attend either the music or the standard curriculum and by their previous music training allowed us to assess their starting features and the effect of music training on a vast pool of cognitive dimensions in the same group of participants.

In what follows we report a longitudinal study based on cognitive tests on preadolescent students of the Negri-Calasanzio Middle School, located in the San Siro district of Milan (Italy). We assessed non-verbal reasoning, language, reading, memory, numerical, and visuo-spatial skills.

As some students had previous music experiences, i.e., private lessons or music laboratory in which they played an instrument during primary school for at least one continuative year, we also took into account this additional variable, grouping the sample by the school curriculum and by the presence or absence of previous music experience. Finally, in our results we also considered the possible influence of parents’ education.

In sum, in this longitudinal study we explored whether the kids who decided to attend the music curriculum show any cognitive advantage with respect to the standard group, on the one hand, and whether the intensive music training can moderate the developmental trajectories of these groups. We expected that the previous musical experience and perhaps the familial socio-cultural status could predict an overall better cognitive performance: yet, it remained a matter of empirical evaluation whether music training could have a further boosting effect in promoting cognitive maturation showing a group-by-time interaction effect and whether this was a generalized one or a specific one.

## Materials and Methods

### Participants

All the participants were recruited during the school years 2014/2015, 2015/2016, and 2016/2017 at the Negri-Calasanzio Middle School of San Siro, Milan.

Students were enrolled in the study after obtaining written informed consent from the parents.

During the 3 years of study, a total of 351 students belonging to all classes of the institute were tested. To avoid potential confounds, in the following analyses we included only participants who never failed their finals, who did not received a prior diagnosis of a learning disability, who underwent the first evaluation at 6^th^ grade, corresponding to the first year of middle school in Italy, and who participated in the study in both the 6^th^ and the 7^th^ grade (Table 1). None of the participants had a medical history of neurological, developmental or psychiatric disorders. After this selection, we obtained a sample of 128 students (56 males and 72 females, see Table 1 for more details).

**TABLE 1:** Distribution of the participants along the 3 years of the study.

	**6^th^ Grade_(MG/SG) T0_**	**7^th^ Grade _(MG/SG) T1_**
*1^*st*^ year of testing*	60 _(38/22)_	
*2^*nd*^ year of testing*	68 _(34/34)_	60 _(38/22)_
*3^*rd*^ year of testing*		68 _(34/34)_
*Total*	128 _(72/56)_	128 _(72/56)_

*The group size is reported in brackets.*

During a preliminary interview, we asked each student about his eventual previous music experience (further details can be found in Supplementary Table 2), investigating whether they had ever had instrumental music training in the years of primary school. We considered as relevant previous music experience a continuative (at least 1 year) instrumental learning experience during private lessons or specific music laboratories offered by the primary school. Due to the variety of experiences reported (age of starting and ending, eventual participation to both private and group lessons and so on…) it was impossible to use more detailed information on previous music experience. This is why we preferred to classify this information using a categorical variable and, as a consequence, to group the sample on the basis of previous music experience and of the choice of the school curriculum. This approach led us to obtain four groups: the Music Group (MG) without previous music experience, the Music Group with previous music experience (MG_EXP_), the Standard Group without previous music experience (SG) and the Standard Group with previous music experience (SG_EXP_). In each group the age of the participants ranged between 10 and 14 years, as summarized in Table 2.

**TABLE 2:** Demographic information and performances (mean, SD) at the cognitive tests in the four groups of students.

	**MG**			**MG_EXP_**			**SG**			**SG_EXP_**		
	***N***	***Mean***	***SD***	***N***	***Mean***	***SD***	***N***	***Mean***	***SD***	***N***	***Mean***	***SD***
**6^th^ grade**												
*Age*	30	11.40	0.35	42	11.31	0.28	44	11.34	0.33	12	11.36	0.35
*Short story (delayed recall)*	30	26.63	4.26	42	26.86	3.90	44	23.21	4.56	12	25.54	4.39
*Matrix reasoning*	30	24.63	2.70	42	24.67	2.91	44	23.57	3.43	12	23.25	4.37
*Coding*	30	51.43	8.08	42	52.69	8.05	44	50.11	7.88	12	50.25	5.63
*Digit forward*	30	5.77	1.04	42	5.71	1.17	44	5.36	0.78	12	5.92	1.00
*Digit backward*	30	4.50	1.20	42	4.62	1.15	44	4.00	1.06	12	4.42	1.00
*Corsi Block test*	30	5.50	0.73	42	5.50	0.99	44	5.34	0.86	12	5.58	0.67
*Phonemic fluency*	30	33.00	8.93	42	35.69	9.84	44	30.05	7.81	12	33.42	10.18
*Semantic fluency*	30	56.33	12.72	42	60.31	10.25	44	52.91	10.46	12	58.00	13.05
*Spoonerisms (sec.)*	30	122.68	67.46	42	130.86	83.10	44	177.47	105.16	12	152.31	114.57
*Spoonerisms (error rate)*	30	3.48	2.57	42	3.55	2.89	44	4.58	3.15	12	4.46	3.46
*Word reading (sec.)*	30	75.38	14.10	42	76.52	20.23	44	86.72	28.75	12	78.66	20.42
*Word reading (error rate)*	30	1.90	1.84	42	1.93	1.69	44	2.91	2.80	12	2.50	2.47
*Pseudo-word reading (sec.)*	30	55.59	9.51	42	61.27	19.64	44	62.43	20.66	12	58.24	18.15
*Pseudo-word reading (error rate)*	30	3.47	3.88	42	4.05	3.99	44	5.02	3.74	12	4.00	2.95
*Text reading (sec.)*	30	127.16	27.35	42	126.67	32.41	44	143.82	55.22	12	130.70	34.32
*Text reading (error rate)*	30	6.00	5.21	42	6.45	4.62	44	9.27	6.46	12	5.77	2.76
*Calculation (sec.)*	30	86.38	50.57	42	87.08	53.34	44	130.62	84.85	12	105.92	59.69
*Calculation (error rate)*	30	1.50	1.61	42	1.76	1.76	44	4.16	2.94	12	2.42	1.68
*Neologisms’ manipulation*	30	16.31	2.69	42	16.33	2.63	44	13.93	3.38	12	13.88	4.11
*Active-to-passive transformation*	30	3.17	2.09	42	4.01	1.53	44	2.67	1.50	12	3.13	1.42
**7^th^ grade**												
*Age*	30	12.57	0.29	42	12.48	0.29	43	12.56	0.31	12	12.48	0.29
*Short story (delayed recall)*	30	27.63	3.95	42	28.64	3.52	43	24.77	5.21	12	25.54	4.13
*Matrix reasoning*	30	25.63	2.63	42	26.19	2.56	43	24.37	3.03	12	24.92	2.68
*Coding*	30	61.60	7.41	42	59.38	11.14	43	59.58	10.46	12	56.92	7.77
*Digit forward*	30	6.23	1.07	42	6.21	1.35	43	5.77	0.92	12	5.92	1.38
*Digit backward*	30	5.23	1.36	42	5.14	1.20	43	4.35	1.00	12	4.58	1.08
*Corsi Block test*	30	5.73	1.01	42	6.00	0.99	43	5.44	0.88	12	5.83	0.72
*Phonemic fluency*	30	34.23	7.29	42	37.33	8.01	43	34.51	9.03	12	33.67	10.50
*Semantic fluency*	30	58.47	12.05	42	66.21	11.43	43	54.61	8.75	12	61.67	12.43
*Spoonerisms (sec.)*	30	91.17	56.06	42	92.20	57.53	43	131.80	77.58	12	96.72	62.04
*Spoonerisms (error rate)*	30	2.77	2.13	42	2.66	2.30	43	4.08	2.98	12	3.33	1.98
*Word reading (sec.)*	30	63.32	9.94	42	65.48	15.54	43	70.34	19.68	12	69.01	17.76
*Word reading (error rate)*	30	1.30	1.47	42	0.95	1.10	43	2.49	2.32	12	1.92	1.56
*Pseudo-word reading (sec.)*	30	46.56	8.86	42	51.79	14.76	43	51.63	16.43	12	55.70	18.13
*Pseudo-word reading (error rate)*	30	2.93	2.83	42	3.33	2.83	43	4.12	2.88	12	3.17	2.44
*Text reading (sec.)*	30	100.56	18.80	42	104.64	24.31	43	112.54	32.31	12	111.37	25.48
*Text reading (error rate)*	30	4.20	2.28	42	3.29	2.51	43	5.49	3.57	12	3.67	3.34
*Calculation (sec.)*	30	59.25	37.41	42	57.56	33.19	43	88.25	50.15	12	85.30	80.53
*Calculation (error rate)*	30	1.77	1.43	42	1.21	1.41	43	2.72	2.59	12	2.42	2.71
*Neologisms’ manipulation*	30	17.47	2.41	42	17.50	2.14	43	15.13	2.98	12	17.00	2.79
*Active-to-passive transformation*	30	4.63	1.83	42	4.95	1.59	43	3.37	1.94	12	4.96	1.66

*MG, Music Group; MGexp, Music Group with previous music experience; SG, Standard Group; SGexp, Standard Group with previous music experience. [Sample size (*N*), mean score and standard deviation (*SD*) of the variables collected at the 6^*th*^-grade (t0) and the 7^*th*^-grade (t1)].*

The study was approved by the Ethical Committee of the University of Milano-Bicocca (prot. num. 188) and by the headmaster and the teaching staff of Negri-Calasanzio Middle School.

#### Music Training in the Two Curricula

The Negri-Calasanzio School where we performed our research offers two different curricula, a Standard Curriculum and a Music Curriculum. In the Standard curriculum, students attend to the regular school program and to the canonical 2 h of music class per week, where, at the most, they are thought to play a recorder (the Italian “flauto dolce”).

In the Music curriculum, students, besides the music training given to all other students, receive two additional hours of music training in ensembles and an individualized hour of training on the instrument of their choice (e.g., guitar, cello, violin, piano, saxophone, or drums). The students of the Standard Curriculum attend instead to artistic and scientific laboratory activities while their peers receive their extra-hours of music training. Accordingly, while the overall timetable of educational activities was balanced across groups, the Standard Curriculum group was not totally naïf to music training, rather they were submitted to standard low intensity training typical of Italian non-music-oriented- middle schools.

### Materials and Procedure

Students were evaluated by means of a selected pool of standardized cognitive tests and by means of cognitive tasks in the domain of non-verbal reasoning, speed of processing, verbal long-term memory, short-term and working memory, lexical access, phonological awareness, reading skills, calculation skills, and morpho-syntactic awareness. The tests selected to be included in the cognitive assessment were part of the Italian version of WISC-IV (Wechsler, 2003) or were extracted from different batteries for the assessment of specific cognitive abilities like reading, phonological skills, morpho-syntactic skills and math skills. Unfortunately, not all the selected measures (see the spoonerisms or the calculation test) were standardized for this age, in which is rather difficult to find specific test addressed to phonological and language skills, or find alternatives to subtests of the WISC-IV.

All participants were assessed during individual sessions; each student underwent two testing sessions per year of about 1 h, whit about a 1-week interval between the first and the second assessment, to complete the entire psychological battery.

During the same testing sessions, we also evaluated implicit and explicit measures of personality, self-esteem, empathy, racial prejudice and tolerance: the results of these tests will be further investigated in a separate work.

#### Non-verbal Reasoning

It was tested using the *Matrix Reasoning* subtest from the Wechsler Intelligence Scale for Children-IV (WISC-IV; Wechsler, 2003).

#### Speed Processing

It was assessed using the *Coding* subtest from the WISC-IV (Wechsler, 2003).

#### Verbal Long-Term Memory

It was evaluated with the immediate and delayed *Recall of a Short Story Test* (Scarpa et al., 2006). Performance was measured as follows: 1 point was assigned for each conceptual cluster if all words were reported exactly as they were heard, and 0.5 points were assigned if the concept retrieved was correct, yet this was done using different words^6^.

#### Short-Term Memory and Working Memory

Short-term memory and working memory were evaluated using the Digit Span (forward and backward) subtest of the Working Memory Index of the WISC-IV (Wechsler, 2003). Visuo-spatial short-term memory was assessed using the Corsi Block Test (Bisiacchi et al., 2005).

#### Lexical Access

It was tested using a Verbal Fluency test (Bisiacchi et al., 2005) both with phonemic and semantic cues.

Phonological awareness was assessed using the *Spoonerisms* subtest of the “Battery for the evaluation of meta-phonological abilities” (Marotta, 2008).

#### Reading Skills

Single words and pseudo-words reading were assessed by means of the DDE-2 Battery (Battery for the assessment of Developmental Dyslexia and Dysorthographia-2; Sartori et al., 2007) where accuracy and reading speed are measured.

*Text reading* was assessed by means of a short-story titled “The Ecologic Disaster” consisting in 610 words selected from the *MT advanced reading battery* (Cornoldi et al., 1998).

#### Arithmetic Skills

Arithmetic skills were tested using the *Calculation* subtest from the “Battery for the assessment of arithmetic skills” (Cornoldi and Cazzola, 2004).

#### Morpho-Syntactic Awareness

Two subtests from the “battery for morphological and morpho-syntactic skills” were used (Co.Si.Mo, Milani et al., 2005). The *subtest 2 a-b-c*, requires the participant to insert the correct flexed form of suggested neologisms in a sentence, with the function of a noun (seven trials), verb (seven trials), or adjective (eight trials). The raw score was calculated as the sum of scores assigned to each answer. The maximum possible score was of 22. *Subtest 7* required an active-to-passive transformation of a target sentence and comprises seven sentences. Accuracy was recorded as the raw score.

The average scores of each test divided by group are reported in Table 2.

### Data Analysis

Statistical analyses were performed in the statistical programing environment *R* (R Core Team, 2018).

As the first step (1) we reduced the data dimensionality using a principal component approach and evaluated whether the factorial structure was stable across time. The PCA-derived newly identified variables were the dependent variables of further analyses used to assess (2) the impact of parental education (3) the longitudinal effect of specific music training.

#### Principal Component Analysis

We ran a principal component analysis (PCA) to reduce the number of variables considered and to group them into higher order cognitive dimensions. As the observed variables are by definition highly correlated, we chose an oblique rotation, in particular, we applied an Oblimin Rotation with Kaiser link (see Costello and Osborne, 2005 for a review). The PCA was run both on T0 and T1 data to verify whether variables were organized in the same latent structure notwithstanding the biological development. The congruence between the two factorial structures was tested through Tucker’s Phi (Tucker, 1951) using the *factor.congruence* routine available in the “psych” *R* package (Revelle, 2004). Once obtained information about the equivalence of the factorial structures extracted at T0 and T1, the factorial weights extracted from T0 were applied to obtain also the factorial scores at T1 by means of a regressive model. These scores were used as variables for the following analyses.

#### Impact of Parental Education on Cognitive Profile at T0 and Curriculum Choice

As first step, a chi-squared test was run to investigate whether the four groups were matched for each parental level of instruction. Then, the influence of parents’ education (Father vs. Mother), rated in 3 levels (1 = primary/middle school, 2 = high school, 3 = university), on each cognitive factor was investigated with 3^∗^2 generalized linear models (GLMs). These were fitted according to the results of a preliminary evaluation of data distribution. The evaluation of data distribution was made by means of graphic analyses and by an *ad hoc* R-routine designed to test the fit between the observed data and the main probability distributions (see Supplementary Material for more details). For example, if the data distribution was positively skewed and the probability distribution with the best fit to the empirical data was the Gamma distribution, we applied a linear transformation of the data to transpose all the values to the positive axis. This allowed us to apply a General Linear Model with a Gamma probability distribution and an “inverse” link function, if needed.

#### Impact of Previous Music Experience (at T0) and Longitudinal Effect of Specific Music Training

To evaluate the cognitive development trajectories of the Music and Standard Groups with and without previous music experience (MG_EXP_, SG_EXP_, MG, SG respectively), we run a series of general linear mixed effect models (GLMMs) on each cognitive factor, using the “lme4” R package (version 1.1-5, Bates et al., 2014). The fixed effects were modeled to test the main effect of Group, the main effect of Time (T0, T1) and their second level interactions, while the Subject ID was considered as clustering factor to model random intercept. Moreover, we also considered the potential influence of the variable “parents’ nationality” (classified as 1 = both parents were Italian, 2 = one of the parents was Italian, 3 = none of the parents was Italian) as nested variable; to do so, we estimated the Intraclass Correlation Coefficient (ICC) for this variable on each cognitive factor (details are reported in Supplementary Table 3). On the basis of this preliminary evaluation, the parents’ nationality was included as further clustering factor only for the first component extracted by the PCA (ICC = 0.37 [0.11–0.96]).

## Results

### Data Reduction and Stability of Factorial Structure

Using a PCA and the scree-plot method (see Supplementary Figure 1), we identified four linear components at T0 with a minimum eigenvalue (i.e., the eigenvalue of the 4^th^ component = 1.103). The same procedure was applied to the data collected at T1 (obtaining a minimum eigenvalue = 1.110). Each factor extracted at T0 was highly correlated with the corresponding factor extracted at T1 [Tuker’s Phi test: Factor 1 (Phi = 0.87); Factor 2 (Phi = 0.96); Factor 3 (Phi = 0.85); Factor 4 (Phi = −0.85)]. The weights extracted at T0 were then used to obtain factorial scores both at T0 and at T1. Only the variables with loadings ≥ |0.3| were considered to identify the cognitive dimension associated with each factor (see Table 3).

**TABLE 3:** Saturation matrix obtained by the principal component analysis (PCA).

	**General cognitive abilities**	**Speed of linguistic elaboration**	**Accuracy in reading and memory tests**	**Visuo-spatial and numerical skills**
*Short story delayed recall*	**−0.371^∗^**	0.044	**−0.444^∗^**	0.100
*Matrix reasoning*	**−0.624^∗^**	0.223	–0.082	–0.020
*Coding*	0.149	–0.027	0.067	**0.748^∗^**
*Digit forward*	–0.267	–0.217	–0.209	0.136
*Digit backward*	**−0.334^∗^**	–0.125	**−0.302**	0.216
*Corsi Block test*	0.037	0.049	–0.178	**0.700^∗^**
*Phonemic fluency*	**−0.643^∗^**	–0.233	0.216	0.007
*Semantic fluency*	**−0.716^∗^**	–0.156	0.224	0.101
*Spoonerisms (sec.)*	**0.478^∗^**	**0.437^∗^**	0.180	0.082
*Spoonerisms (error rate)*	**0.413^∗^**	0.171	**0.438^∗^**	0.276
*Word reading (sec.)*	0.080	**0.902^∗^**	–0.035	–0.076
*Word reading (error rate)*	–0.117	**0.467^∗^**	**0.575^∗^**	–0.021
*Pseudo-word reading (sec.)*	–0.147	**0.934^∗^**	0.001	0.004
*Pseudo-word reading (error rate)*	–0.105	0.031	**0.874^∗^**	–0.031
*Text reading (sec.)*	0.066	**0.877^∗^**	0.083	0.031
*Text reading (error rate)*	0.033	–0.030	**0.816^∗^**	–0.084
*Calculation (sec.)*	**0.399^∗^**	0.246	–0.082	**−0.428^∗^**
*Calculation (error rate)*	**0.458^∗^**	–0.007	0.092	**−0.462^∗^**
*Neologisms’ syntactic manipulation*	**−0.667^∗^**	0.041	–0.137	–0.004
*Active-to-passive transformation*	**−0.682^∗^**	–0.038	–0.097	–0.170

*^∗^Variables with a saturation value ≥ |0.3|.*

The four factors were labeled as follows:

– ***Factor 1:* General Cognitive Abilities**; this factor had substantial loadings from tests requiring reasoning, syntactic linguistic skills, general processing speed, memory.
– ***Factor 2:* Speed of Linguistic Elaboration**; here the contribution was from the speed measures of tests like reading, phonological and morpho-syntactic skills.
– ***Factor 3:* Accuracy in Reading and Memory tests**; this factor had substantial loadings from reading and memory tests as far as the accuracy was concerned.
– ***Factor 4:* Visuo-spatial and numerical skills**; here the contribution was from tests like the digit symbol of the WISC-IV, the Corsi Block-tapping test (short-term) and calculation skills.

### Impact of Parental Education on Cognitive Profile at T0 and Curriculum Choice

The chi-squared test on the parental education data showed that the groups were not matched either for education of the father (X^2^_(6)_ = 28.3, *p* < 0.001), or for education of the mother (X^2^_(6)_ = 18.76, *p* = 0.004; see Table 4 for full-detailed contingency tables).

**TABLE 4:** Frequency table of parents’ educational levels, differentiated for father and mother, in each group.

**Educational Level**		**1**	**2**	**3**	
		**Primary/Middle**	**High**	**University**	**Total**
		**School**	**School**		
*Father*					
	*SG*	14	23	7	44
	*SG*_EXP_	1	4	7	12
	*MG*	1	17	12	30
	*MG*_EXP_	4	12	26	42
*Mother*					
	*SG*	9	25	10	44
	*SG*_EXP_	0	5	7	12
	*MG*	2	14	14	30
	*MG*_EXP_	3	12	27	42

In both cases the participants included in the Music Group with previous music experience (MG_EXP_) had parents with a higher level of education.

However, the level of parental education was not significantly related to any of the cognitive factors measured at T0 (see Table 5).

**TABLE 5:** Statistical tests and significance of the Main effects and interactions emerged by the GLMs run on each cognitive factor identified by PCA.

**Cognitive factor**	**Fixed effect**	**X^2^**	**df**	***p***	**η^2^**	**R^2^**
*General cognitive abilities*	*Father’s Education*	4.58	2	0.1	0.064	0.104
	*Mother’s Education*	1.68	2	0.43	0.013	
	*Father’s Education^∗^ Mother’s Education*	3.70	4	0.44	0.028	
*Speed of linguistic elaboration^#^*	*Father’s Education*	1.76	2	0.41		0.101
	*Mother’s Education*	5.58	2	0.06		
	*Father’s Education^∗^ Mother’s Education*	4.09	4	0.39		
*Accuracy in reading and memory tests^#^*	*Father’s Education*	2.34	2	0.30		0.78
	*Mother’s Education*	3.39	2	0.18		
	*Father’s Education^∗^ Mother’s Education*	0.89	4	0.92		
*Visuo-spatial and numerical skills*	*Father’s Education*	1.58	2	0.45	0.007	0.035
	*Mother’s Education*	2.13	2	0.34	0.017	
	*Father’s Education^∗^ Mother’s Education*	1.42	4	0.83	0.012	

*^#^Generalized linear model with a family Gamma distribution and inverse link function.*

### Longitudinal Effect of Intensive Music Training

From the graphic exploration of data distribution we identified 7 and 4 (repeated-measures) outliers in the Factor 2 and in the Factor 3 respectively. These data were removed in order to normalize the data distribution. The data of the four cognitive factors, identified by means of the PCA, were then analyzed using a series of GLMMs to test the effect of Group, of Time and their second level interactions.

**FIGURE 1:**
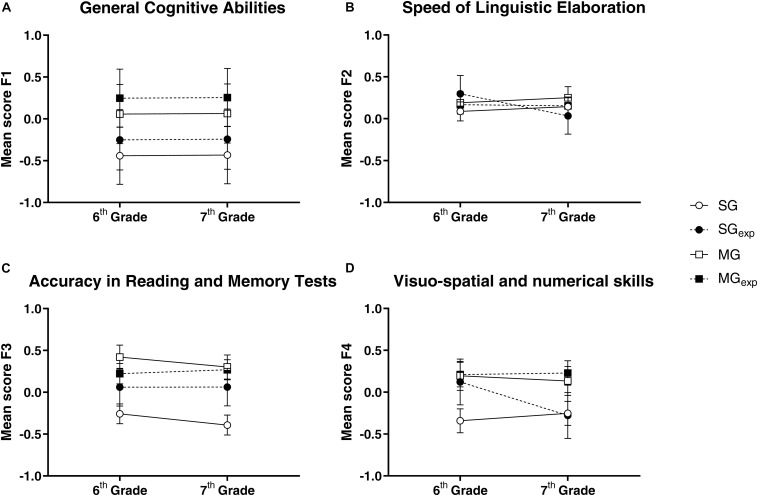
Mean factor scores collected in the MG and SG with and without previous music experience at T0 and T1. Error-bars represents mean standard errors. The average factor scores reported for factors 1, 2, and 3 were multiplied by –1 to facilitate results interpretation. Accordingly, for all factors in this figure, a higher score corresponds to a better mean performance. In the panel **A** mean factor scores of factor 1 (General Cognitive Abilities) are shown; in the panel **B** mean factor scores of factor 2 (Speed of Linguistic Elaboration) are shown; in the panel **C** mean factor scores of factor 3 (Accuracy in Reading and Memory Tests) are shown; in the panel **D** mean factor scores of factor 4 (Visuo-spatial and numerical skills) are shown.

The GLMMs revealed a significant main effect of group for the General Cognitive Abilities (X^2^_(3_,_255)_ = 16.38, *p* < 0.001; see Figure 1A), for the Accuracy in Reading and Memory Tests^7^ (X^2^_(3_,_248)_ = 21.12, *p* ≤ 0.001; see Figure 1C), and for the Visuo-spatial and Numerical Skills (X^2^_(3_,_255)_ = 9.58, *p* = 0.02; see Figure 1D). No main effects of Time (6^th^-grade and 7^th^-grade) were found, nor a Group-by-Time interaction (see Table 6 for more details). We further explored the effect of the group by means of *post hoc* comparisons (FDR-corrected; Table 7). In general, the students included in the MG_EXP_ group outperformed their peers in the level of General Cognitive Abilities, of Accuracy in Reading and Memory Tests. There was also a tendency in the Visuo-spatial and Numerical Skills components.

**TABLE 6:** Statistical tests and significance of the fixed effects emerged by the GLMMs run on each cognitive factor.

**Cognitive factor**	**Fixed Effect**	**X^2^**	**df**	***p***	**ICC_random_ [lower-upper]**	***f*^2^**
*General cognitive abilities^+^*	*Group*	16.3	3	<0.001^∗∗∗^	ID = 0.85 [0.76-0.87]	0.088
	*Class*	0.02	1	0.86	PN = 0.37 [0.11-0.96]	
	*Group^∗^Time*	1.89	3	0.59		
*Speed of linguistic elaboration^∧^*	*Group*	0.40	3	0.93	ID = 0.82 [0-76-0.87]	0.007
	*Class*	0.00	1	0.92		
	*Group^∗^Time*	5.93	3	0.11		
*Accuracy in reading and memory tests ^#^*	*Group*	21.12	3	<0.001^∗∗∗^	ID = 0.67 [0.57-0.76]	0.141
	*Class*	0.93	1	0.33		
	*Group^∗^Time*	1.91	3	0.58		
*Visuo-spatial and numerical skills*	*Group*	9.58	3	0.02^∗^	ID = 0.6 [0.47-0.7]	0.064
	*Class*	0.04	1	0.83		
	*Group^∗^Time*	3.17	3	0.36		

*Interactions are not reported as the best fitting models did not contributed to the better fitting of the data. *^+^*Parent’s nationality was used as random intercept. ^∧^14 observations were rated as outliers and were removed (see Supplementary Material). *^#^*7 observations were rated as outliers and were removed (see Supplementary Material). ^∗∗∗^*p* < 0.001, ^∗∗^*p* < 0.01, and ^∗^*p* < 0.05.*

**TABLE 7:** *Post hoc* comparisons (FDR-corrected) conducted on the Group variable, taking the Time as a fixed parameter.

		**Group-**	**Value**	**df**	**X^2^**	***p***
		**comparison**				
*General*	*T0*	SG-SG_EXP_	0.061	1	0.049	0.825
*cognitive*		SG-MG	0.492	1	5.943	**0.059^∗^**
*abilities*		SG-MG_EXP_	0.643	1	11.782	**<0.001^∗∗∗^**
		SG_EXP_ –MG	0.431	1	2.278	0.225
		SG_EXP_ -MG_EXP_	0.582	1	4.611	0.076
		MG- MG_EXP_	0.151	1	0.578	0.488
	*T1*	SG-SG_EXP_	0.232	1	0.711	0.488
		SG-MG	0.456	1	5.101	0.072
		SG-MG_EXP_	0.730	1	15.125	**<0.001^∗∗∗^**
		SG_EXP_ –MG	0.224	1	0.617	0.488
		SG_EXP_ -MG_EXP_	0.498	1	3.377	0.132
		MG- MG_EXP_	0.274	1	1.894	0.253
*Accuracy in*	*T0*	SG-SG_EXP_	0.318	1	1.576	0.359
*reading and*		SG-MG	0.678	1	13.244	**<0.001^∗∗∗^**
*memory*		SG-MG_EXP_	0.479	1	8.006	**0.014^∗∗^**
*tests*		SG_EXP_ -MG	0.360	1	1.822	0.354
		SG_EXP_ -MG_EXP_	0.161	1	0.399	0.575
		MG- MG_EXP_	–0.199	1	1.109	0.438
	*T1*	SG-SG_EXP_	0.454	1	3.188	0.178
		SG-MG	0.694	1	13.739	**<0.001^∗∗∗^**
		SG-MG_EXP_	0.661	1	15.236	**<0.001^∗∗∗^**
		SG_EXP_ -MG	0.241	1	0.816	0.489
		SG_EXP_ -MG_EXP_	0.207	1	0.664	0.498
		MG- MG_EXP_	–0.034	1	0.032	0.858
*Visuo-*	*T0*	SG-SG_EXP_	–0.464	1	2.263	0.265
*spatial and*		SG-MG	–0.537	1	5.723	0.075
*numerical*		SG-MG_EXP_	–0.553	1	7.311	0.075
*skills*		SG_EXP_ -MG	–0.073	1	0.050	0.944
		SG_EXP_ -MG_EXP_	–0.088	1	0.081	0.944
		MG- MG_EXP_	–0.016	1	0.005	0.944
	*T1*	SG-SG_EXP_	0.026	1	0.007	0.944
		SG-MG	–0.387	1	2.961	0.243
		SG-MG_EXP_	–0.482	1	5.522	0.075
		SG_EXP_ -MG	–0.413	1	1.629	0.346
		SG_EXP_ -MG_EXP_	–0.508	1	2.683	0.243
		MG- MG_EXP_	–0.095	1	0.175	0.944

*^∗∗∗^*p* < 0.001, ^∗∗^*p* < 0.01, and ^∗^*p* < 0.05.*

The lack of significant Group-by-Time interaction suggests that most of the main effects observed are related to natural predisposition and, possibly, earlier environmental effects that remain relatively stable, at least for what concerns this specific age and our specific time-window (i.e., 1 year); this issue will be discussed in details later on.

## Discussion

There is a long tradition of studies assessing whether music training has an effect on the development of cognitive skills: the issue has been evaluated primarily by searching for differences between musicians and non-musicians (Brochard et al., 2004; Franklin et al., 2008; Jakobson et al., 2008; Tierney et al., 2008; Groussard et al., 2010; George and Coch, 2011). There is also a growing body of studies that addressed this issue using a longitudinal approach in children (Schellenberg, 2004, 2006; Tierney et al., 2008; Corrigall and Trainor, 2011; Moreno et al., 2011a; Roden et al., 2012).

Taken together, these studies indicate a cognitive boosting effect of music training.

However, this conclusion was heavily influenced by empirical findings based on retrospective cross-sectional studies: these, by their nature, cannot establish firm causal links between music training and cognitive development. This point has been stressed by Schellenberg (2004, 2006, 2011) who also raised the doubt that certain cognitive advantages in musicians should be considered as indices of a natural predisposition for music and its learning rather than as the consequence of intensive music training. Accordingly, musicians would have a natural *gift* that could be boosted by the continuing music training; the same natural predisposition and/or environmental advantage would influence the choice to start the music training itself and the high cognitive challenge that it implies.

Our study was designed to assess these issues in pre-adolescents in what we realistically consider a quasi-experimental setting, given that some of the independent variables were outside our control (e.g., previous music experience; parental education; socio-economical status, assignation to a given curriculum).

While we are not able to disentangle these issues once and for all, it is noteworthy that the superiority that we found in children of the music training groups was not enhanced by 1 year of further intensive music training received in the special music curriculum at school.

### Factorial Structure of the Cognitive Tests on the Entire Sample of Adolescents

The cognitive skills of our participants were assessed by means of an extensive cognitive battery. The tests selected were mainly extracted from the most common clinical protocols for the assessment of learning disorders used in Italy, which comprises: reading and math tests, visuo-spatial and verbal short-term memory evaluation and a general IQ assessment. Non-verbal reasoning has been here assessed by means of some specific subtests of the WISC-IV (Wechsler, 2003)^8^.

As the range of variables collected was a large one, the pool of cognitive data was reduced with a PCA into four components: (1) General Cognitive Abilities (2) Speed of Linguistic Elaboration (3) Accuracy in Reading and Memory tests (4) Visuo-spatial Skills and numerical skills.

A brief comment on the composition of these factors is in order. Factor 1 had weights primarily from tests requiring reasoning and processing speed combined. Interestingly, the reading and phonological skills tests were segregated for their speed and accuracy in separate factors (2 and 3), as one would expect from the well-known independence of reading and reading-related skills from general intelligence.

Also, the fourth factor had interesting features, as it received weights from both visuo-spatial skills and numerical skills tests. This association is not that surprising: a long tradition of studies on the mental number line and the so-called SNARC (spatial-numerical association of response codes) effect connects spatial and numerical cognition (Dehaene et al., 1993). Furthermore, numerical and spatial cognition share similar neurofunctional underpinnings in dorsal parietal cortex and the intra-parietal sulcus, both in humans (Piazza et al., 2004; Swisher et al., 2007), and in monkeys (see Grefkes and Fink, 2005 for a review).

To the best of our knowledge, there is not a prior any similar exploration of the cognitive profile of adolescents using a PCA of a broad test battery. Hence, we are unable to compare the present factorial structure with a similar analysis in the literature. Analyses of the factorial structure of other test batteries (WISC or even the WAIS) are not readily comparable either, given the differences with our battery. Yet, it is worth mentioning that a relatively recent re-assessment of the factorial structure of the WAIS (Gignac, 2005) using a confirmatory factor analysis, suggests that the best fitting model should incorporate a set of nested factors: at the top of the hierarchy a “g -general intelligence- factor,” with three underlying factors representing, respectively, “vocabulary comprehension,” “freedom from distractibility,” and “perceptual organization.”

In any event, what counts here is that our factorial solutions were interpretable and stable over time, not suggesting a qualitative change in the cognitive architecture of our participants over the year when they were under our observation. This consideration further justified the exploration of the data discussed below.

### Effects of Parental Education on the Decision of Joining a Music Curriculum and Cognitive Makeup at T0

According to our data, there was a sizeable effect of parental education on our findings. Indeed, we found that the parents of the “MG_EXP_ group” had overall a better education (see Table 4); however, this factor did not predict in a systematic manner the level of cognitive performance at T0 of our subjects. Still, there was a trend for a significant impact of the level of maternal education over the speed of linguistic elaboration.

The *post hoc* analyses on our main GLMs allowed us to explore any group difference already present at T0. In a nutshell, we found that the students of the Music Group (particularly those with previous music experience) systematically outperformed the students of the Standard Group. The lack of a significant difference between participants of the Music Group with and without previous music experience suggests the existence of a sizeable cognitive advantage behind the decision of joining an additional intensive music training in middle school. However, we believe that this was only one side of the coin as the data on parental education suggest that higher education of the parents is associated with the likelihood of joining the more intensive music program of our music curriculum. Accordingly, overall our observations at T0 suggest that a combination of cognitive features and familiar environment have an impact on the choice of joining a music curriculum.

Indeed, we found that children in the MG_EXP_ group, i.e., the group of children who had a previous music experience during primary school and also decided to attend the Music curriculum in the middle school, come from families with a higher socio-economical status. This suggests that parental pressure may have contributed to the choice of attending the more intense music training both at the primary and at the middle school; this possibility should be further explored and taken into account when planning new school-policies and programs.

### Music Education at School: Too Little and Too Late?

One of our research questions was whether there was an effect of the musical training on the cognitive maturation trajectory of our pre-adolescents or whether it was too late to detect any meaningful effect at this age. Another important question was whether any previous music training, and the relative cognitive profile, were systematically associated with our empirical findings over time. In other words, the question was whether the “damage or the blessing” was already present by the time of our observation. Furthermore, another important question was whether any effect of music training has an impact on skills directly relevant for music performance or rather it generalizes to distant cognitive domains.

The longitudinal design of our study tried to answer these questions, at least for children attending to middle school. In a nutshell, we found that the MG_EXP_ and MG groups were superior for their General Cognitive Abilities and Accuracy in Reading and Memory tests from outset (at T0) and that these differences were maintained over time (at T1) with no further interactions. This means that no effect of the more intense music practice was observed in this time window and, thus, the cognitive maturation of our students was not specifically affected by the more intense music training or the music curriculum.

A similar interpretation has been drawn from previous correlational studies, like the one of Forgeard et al. (2008) who found better verbal ability and non-verbal reasoning performances in children trained for at least 15 months with music: yet, the possibility of pre-existing cognitive differences between trained participants and controls could not be excluded.

As said, it was impossible to randomize the assignation of our children to the two curricula for obvious ethical reasons as this would have had implications for 3 years of middle school. Yet, even when assignation to experimental and control groups was randomized for a 6-week music treatment in pre-schoolers, as in Mehr et al. (2013), no specific boosting effect could be observed of music training in spatial-navigation, reasoning, visual form analysis, numerical discrimination or receptive vocabulary.

Our results are at variance with those reported by Hyde et al. (2009) and Habibi et al. (2018) who found no difference in the cognitive profile of their participants before half of them received by their choice music training (e.g., key-board lessons or violin lessons): differences in the recruitment criteria and in the age of the participants (our mean age: 11; the cited studies 6–7 years) may explain these discrepancies. One may speculate that younger children have little say compared to their parents in the decision to start music, while pre-adolescents may follow more overtly their predispositions in joining or not joining a music curriculum depending on how easy music is felt by them. These factors may have contributed to the observation of no differences before training in younger children while, as we show, by the time children become pre-adolescents and join a music curriculum, they tend to have superior cognitive performance compared with their classmates.

To summarize, the pattern of results that we describe suggests that the differences observed at the 6^th^ grade (T0) were probably due to the combination of familial status, previous music experience and, maybe, also to a pre-existing cognitive advantage for these students who chose the music curriculum. We can just confirm that in the time span assessed (i.e., after 1 year of intensive music training) this advantage was maintained, on average.

Our results represent the empirical demonstration of the possible bias associated with retrospective studies, at least in this specific field of research: as shown by our data, before attributing to music training the “power” of enhancing the developmental trajectory of a specific cognitive ability, one needs to assess the cognitive profile of the children involved at the onset.

In the same vein, the effect of previous music training should also be taken into account. According to our data, the students in the MGs had better performances regardless of whether they had or not a previous music experience. These observations suggest that students who chose to start the study of an instrument had, in general, a cognitive advantage, at least at this point in their development.

Further studies are needed with larger samples followed up for longer periods (Zuk and Gaab, 2018). Ideally, the first evaluation should occur during either the preschool age, or the primary school and repeated observations should be collected until the end of secondary school. It is also worth recalling that some of the advantages of students in the music curriculum could be accounted for by the family socioeconomic status^9^, a factor that is difficult to control for in a quasi-experimental setting. This factor was associated with the likelihood of children joining the music curriculum rather than with their level of cognitive performance; this should not be that surprising as parental factors do not have a deterministic impact on the level of cognitive performance of the off-springs.

To conclude, for the time being, 1-year of intensive music training does not have a meaningful effect on the cognitive maturation of pre-adolescents or at least it cannot surpass the effect of previous experiences and natural predisposition that may eventually lead to embracing music studies also in early childhood. This conclusion leaves open the possibility that a much earlier introduction of systematic music training might have very different effects on cognitive maturation and represent important support for cognitive development.

Further, a word of caution is needed here as the adolescents of the standard curriculum were not deprived of any music training, rather they received the low-dose music training typical of the standard Italian middle school program.

Another important point to be made relates to the potential effects of music training on the maturation of social and emotional skills and mutual tolerance in children of the same age as those considered here. The present findings do not exclude a specific impact on these affective dimensions even at this relatively late time of mental maturation, something that remains to be tested.

Taken together, our findings also imply that, if anything, educational policies and the needed resources should developed and be put in place to promote music training starting from primary school when there is a better likelihood of having sizeable results on cognitive maturation.

## Data Availability Statement

The datasets generated for this study are available on request to the corresponding author.

## Ethics Statement

The studies involving human participants were reviewed and approved by the Ethics Committee of the University of Milano-Bicocca. Written informed consent to participate in this study was provided by the participants’ legal guardian/next of kin.

## Author Contributions

EP, LD, MTG, NS, PS, MP, MG, and MM conceived and designed the study. LD and DC collected behavioral data. DC, MB, MP, and MG performed the statistical analysis. AM and MM coordinated the relations with the school. DC wrote the first draft of the manuscript. MB and EP revised the manuscript. All authors contributed to the study and to the final revision of the manuscript.

## Conflict of Interest

The authors declare that the research was conducted in the absence of any commercial or financial relationships that could be construed as a potential conflict of interest.

## Footnotes

1 A left lateralized planum temporale has been related to pitch discrimination skills and it is considered as a possible anatomic correlate of the *absolute pitch* (see Schlaug et al., 1995; Schlaug, 2015 for a review).

2 It is well-known that phonological competence is a predictor of the development of fluent reading (Vellutino and Scanlon, 1999).

3 There are few exceptions represented by longitudinal studies (e.g., Schellenberg, 2004; Corrigall and Trainor, 2011; Moreno et al., 2011a; Tierney and Kraus, 2014).

4 By definition, the expression quasi-experimental study refers to non-randomized intervention studies. These designs are the “obvious choice” when it is not logistically feasible or ethical to randomly assign participants to experimental conditions like, for example, in the case of our study in which is not possible to decide whether a kid should attend either the music or standard curriculum at school.

5 Ideally one may want to study children as early as possible during their primary school. However, this is practically impossible in Italy as no systematic formal music training is available for children in that age-range.

6 Different scoring systems give 1 point for each word exactly recalled or for the recall of semantic concepts with greater tolerance on word recall precision.

7 Here it is worth noting that possible outliers were identified by means of boxplots and, consequently, removed (the number of outliers removed are reported in Table 6, while details about their identification can be read in a dedicated section of the Supplementary Material).

8 The choice of WISC-IV’s subtests for a longitudinal design is open to discussion: the WISC-IV’s subtests have a relatively low test-retest reliability compared with composite indices (see Watkins and Smith, 2013). However, our choice of these specific subtests was dictated by pragmatic considerations on the time of administration and, most of all, because of the absence of alternative updated and standardized measures for the specific stage of development examined, at least in the Italian context.

9 It is also worth recalling that the socio-economic status was inferred from the parental education. A detailed evaluation of the socio-economic status was beyond what was permitted by our Ethics Committee.
