# Supplementary material for: Music Education at School: Too Little and Too Late? Evidence From a Longitudinal Study on Music Training in Preadolescents
Source: Front Psychol. 2019 Dec 18;10:2704. doi: 10.3389/fpsyg.2019.02704 (PMC6930811; doi:10.3389/fpsyg.2019.02704)

Supplementary Materials

Music education at school: too little and too late?
Evidence from a longitudinal study on music training in preadolescents.

D. Carioti[1,2], L. Danelli[1], M.T. Guasti[1], M. Gallucci[1], M. Perugini[1], P. Steca[1], N. Stucchi[1], A. Maffezzoli[3], M. Majno[4], M. Berlingeri[2,5,6], E. Paulesu[1,7]

[1] Psychology Department, University of Milano-Bicocca, Milano, Italy.

[2] DISTUM, Department of Humanistic Studies, University of Urbino Carlo Bo, Urbino, Italy.

[3] Negri-Calasanzio Middle School, San Siro, Milan, Italy.

[4] SONG onlus - Sistema in Lombardia, Milan, Italy.

[5] Center of Developmental Neuropsychology, ASUR Marche, Area Vasta 1, Pesaro.

[6] NeuroMi, Milan Center for Neuroscience, Italy.

[7] I.R.C.C.S. Galeazzi, Orthopedic Institute Milano, Milan, Italy.

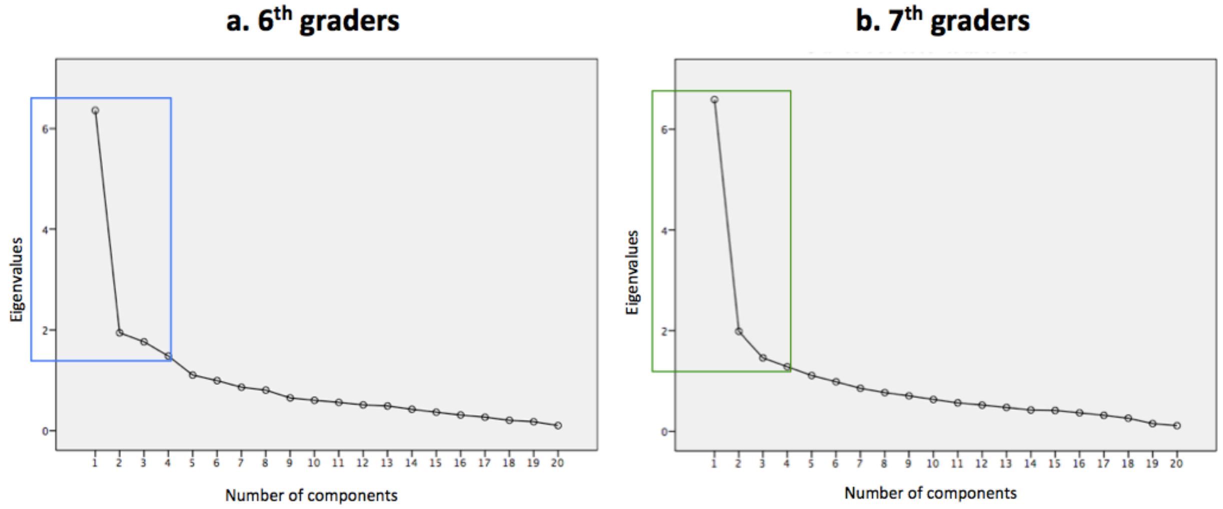

Supplementary Figure 1. Scree-plots used to determine the number of factors to retain in the Principal Components Analysis

Supplementary Table 1. Frequency distribution of parents' nationality across groups.

	Nationality of Parents			
	Both Italians	*One Italian, One foreigner*	*Both foreigners*	
**Groups-by-previous music experience:**				
**SG**	19	6	19	44
**SG_{EXP}**	8	3	1	12
**MG**	23	1	6	30
**MG_{EXP}**	32	6	4	42
Tot.	82	16	30	128

Supplementary Table 2: Years of previous music experience reported by students in the standard and music groups. This variable was used to classify students as with and without previous music experience.

ID	Group	Years of previous music experience
1	MG	2
2	SG	4
3	MG	1
4	MG	2
6	MG	3
11	MG	2
14	MG	2
15	SG	2
18	MG	2
20	MG	3
23	MG	2
24	MG	5
25	SG	5
26	MG	3
28	MG	5
30	MG	3
31	MG	2
32	MG	2
38	SG	3
39	MG	1
41	SG	1
42	SG	5
47	MG	3
50	MG	4
51	MG	6
53	MG	2
55	MG	1
56	MG	1
58	MG	1
66	MG	3
67	MG	2
68	MG	1
69	MG	3
71	SG	2
73	MG	2
76	MG	3
77	SG	3
82	MG	2
83	MG	2
84	MG	7
86	MG	1
88	MG	1
92	MG	1
93	MG	1
100	MG	2

102	SG	1
104	MG	1
105	SG	2
106	MG	2
111	MG	2
115	SG	1
119	MG	2
121	MG	5
127	SG	1

Supplementary Table 3. Intraclass Correlation Coefficient (ICC), used to investigate whether the scores of each factor were clustered on the basis of parents' nationality.

Intraclass Correlation Coefficient	ICC	LowerCI	UpperCI	N	k
Parents' Nationality ~ Factor 1	0.373	0.11	0.96	3	33.21
Parents' Nationality ~ Factor 2	-0.004	-0.02	0.49	3	33.21
Parents' Nationality ~ Factor 3	0.038	-0.011	0.73	3	33.21
Parents' Nationality ~ Factor 4	-0.007	-0.02	0.45	3	33.21

Identification of outliers and of the model with the best fitting to the empirical data

In what follows we report step-by-step the method that we adopted to evaluate the fitting of the empirical data distribution to a Gaussian probability distribution and to identify outliers:

1) we explored the empirical distribution of the factorial scores obtained by the PCA and we checked for the presence of outliers by means of graphic inspection (box-plots);
2) we tested both the normal and the gamma distribution, to identify the probability model with the best fitting to our empirical data.
3) we made these evaluations both for the Generalized Linear Models run at T0 (see the paragraph (2) Impact of parental education on cognitive profile at T0 and curriculum choice in the method section), and for the models including repeated measures at T0 and T1, i.e. the Generalized Linear Mixed models (see the paragraph (3) Impact of previous music experience (at T0) and longitudinal effect of specific music training in the method section).

In what follows, we report in details the procedure applied for each cognitive dimension tested. The reader can find:

1) A detailed description of the empirical data distribution of the factorial score of each dimension tested, together with box-plots and, if necessary, outliers identification
2) The results of the model fitting procedure, according to the following R syntax:
   a. **Testing for normal distribution**:

      qqp(mydata$Factor, "norm")

   b. **Testing for Gamma distribution:**

      - Preliminary data translation on the positive axis:

      mydata$Factor_positive <- mydata$Factor +2

      - Theoretical model fitting:

      gamma <- fitdistr(mydata$Factor_positive, "gamma")

      qqp(mydata$Factor_positive. "gamma", shape = gamma$estimate[[1]], rate = gamma$estimate[[2]])

This information is reported in relation to the separated analyses conducted to test

(a) ***Impact of parental education on cognitive profile at T0 and curriculum choice (GLMs)***

(b) ***Longitudinal effect of intensive music training (GLMMs)***

a) For what concerns the section: ***"Impact of parental education on cognitive profile at T0 and curriculum choice"***

Before running the 3 (parents' education: low-medium-high) *2 (Father-Mother) Generalized Linear Models (GLMs) in which each factor extracted by the PCA was used as dependent variable, the empirical data distribution was evaluated on the basis of the boxplots' inspection and of theoretical model fitting.

Factor 1: General Cognitive Abilities

1.1 empirical data distribution and box-plots

n	*Mean*	*sd*	*Min.*	*Max.*	*Skewness*	*Kurtosis*
128	-0.003	0.997	-1.930	2.640	0.369	-0.490

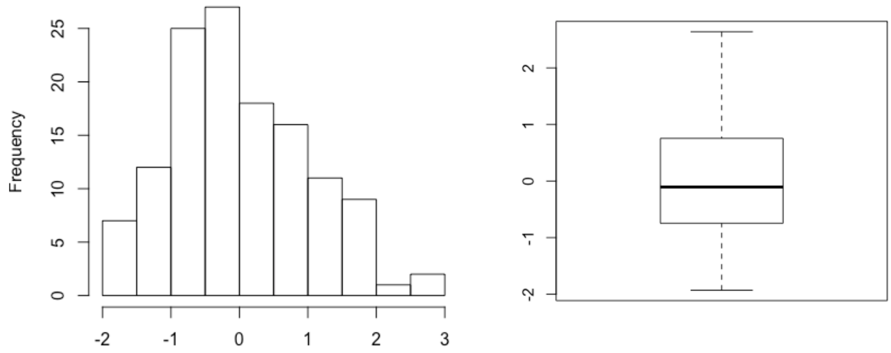

1.2 theoretical model fitting

Normal Distribution:

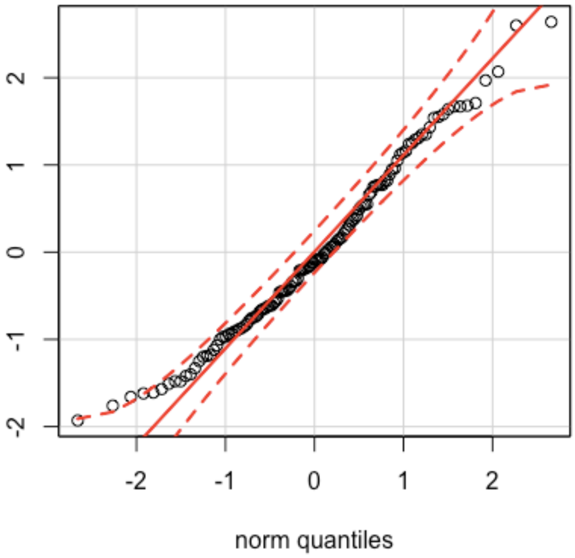

In addition, also the Kolmogorov-Smirnoff[1] test (D = 0.06, p = 0.68) and the Shapiro-Wilk[2] test (W = 0.97, p = .054) confirmed that data were normally distributed.

---

1 The K-S test can be applied to different distributions as it can be read in the following R syntax:
- *ks.test(mydata$Factor1,"pnorm/pgamma")*
[George Marsaglia, Wai Wan Tsang and Jingbo Wang (2003). Evaluating Kolmogorov's distribution. *Journal of Statistical Software*, **8**/18. doi: 10.18637/jss.v008.i18]

Factor 2: Speed of Linguistic Elaboration

2.1 empirical data distribution and box-plots

n	*Mean*	*sd*	*Min.*	*Max.*	*Skewness*	*Kurtosis*
128	-0.008	1.001	-1.540	4.800	1.709	4.273

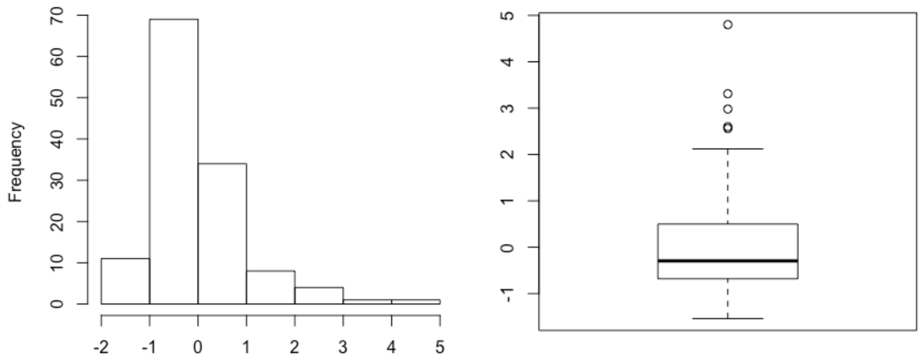

2.2 theoretical model fitting

Normal Distribution: Gamma Distribution:

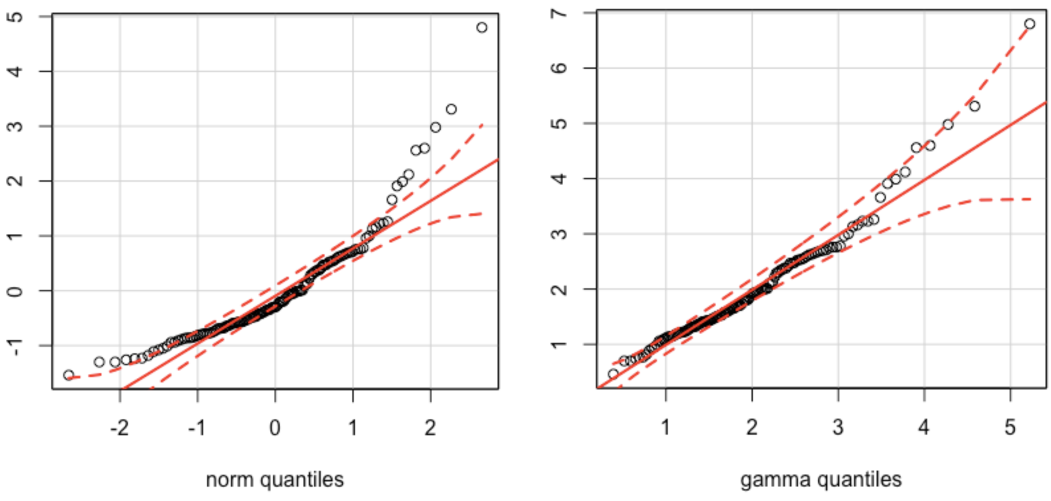

In this case the Gamma has been considered the best fitting distributions on the basis of the above-reported graphs and of the Kolmogorov-Smirnov test for Gamma Distribution ($D = 0.076$, $p = 0.44$). Data distribution was far from being normal:
Kolmogorov-Smirnov test for Normal Distribution: $D = 0.13$, $p = .016$
Shapiro-Wilk test: $W = 0.86$, $p < .001$.

---

[2]The Shapiro-Wilk test for normal distribution has been applied to each factor using the following R syntax:
- *shapiro.test(mydata$Factor1)*
[Patrick Royston (1995). Remark AS R94: A remark on Algorithm AS 181: The W test for normality. *Applied Statistics*, **44**, 547–551. doi: 10.2307/2986146]

Factor 3: Accuracy in Reading and Memory tests

3.1 empirical data distribution and box-plots

n	*Mean*	*sd*	*Min.*	*Max.*	*Skewness*	*Kurtosis*
128	-0.002	0.995	-1.760	3.960	1.239	2.682

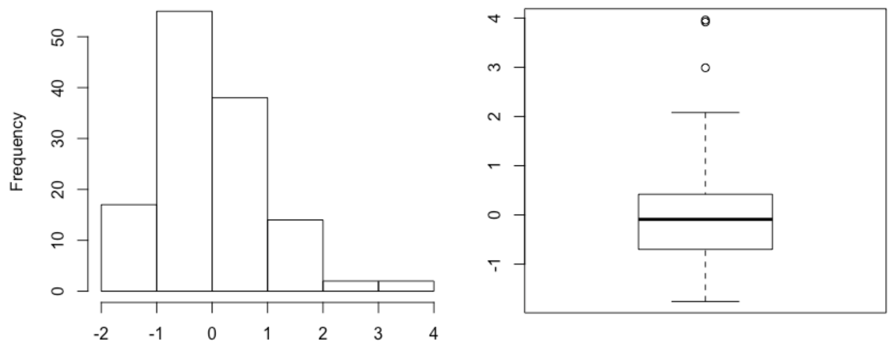

3.2 theoretical model fitting

Normal Distribution: Gamma Distribution:

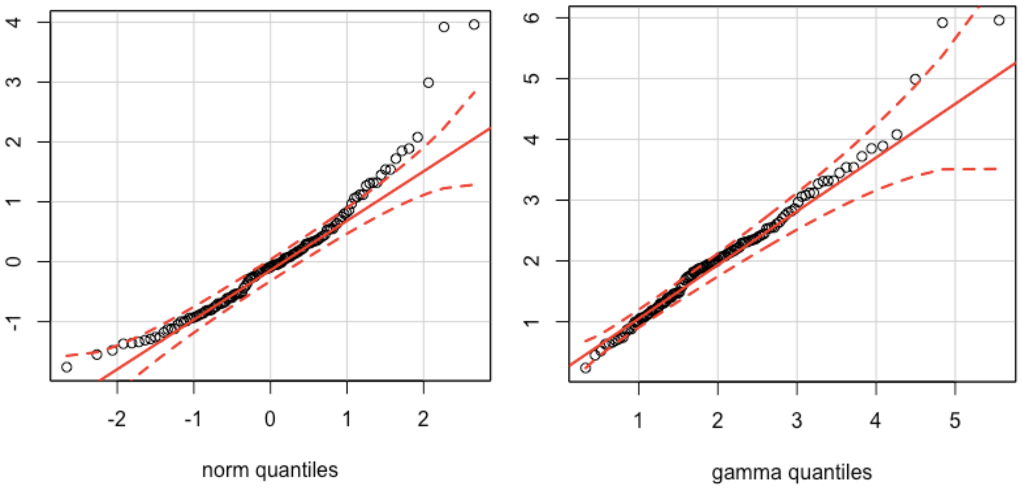

In this case, even though the Kolmogorov-Smirnov test for Normal Distribution was not significant (D = 0.093, p = 0.21), the Shapiro-Wilk test (W = 0.92, p < .001), the descriptive statistics and the graphs indicated a positive skewness. On the contrary, the Kolmogorov-Smirnov test for Gamma Distribution was not significant (D = 0.053, p = 0.85) confirming the results represented in the graph (the right panel). For this reason the GLM was fitted using a Gamma distribution with Inverse Link function.

Factor 4: Visuo-spatial and numerical skills

4.1 empirical data distribution and box-plots

n	*Mean*	*sd*	*Min.*	*Max.*	*Skewness*	*Kurtosis*
128	0.009	0.995	-2.350	2.470	-0.075	-0.301

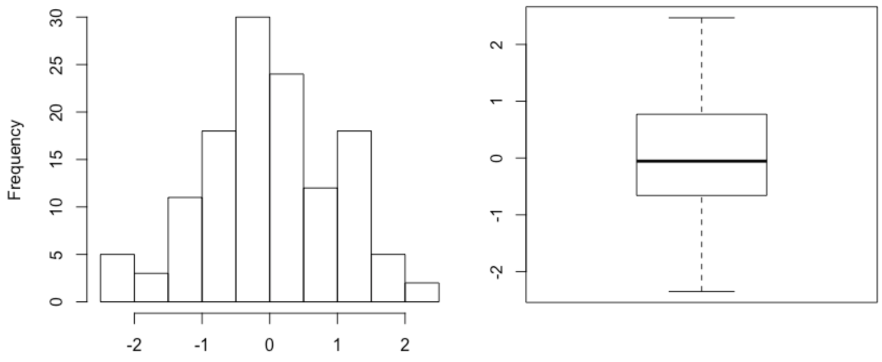

4.2 theoretical model fitting

Normal Distribution:

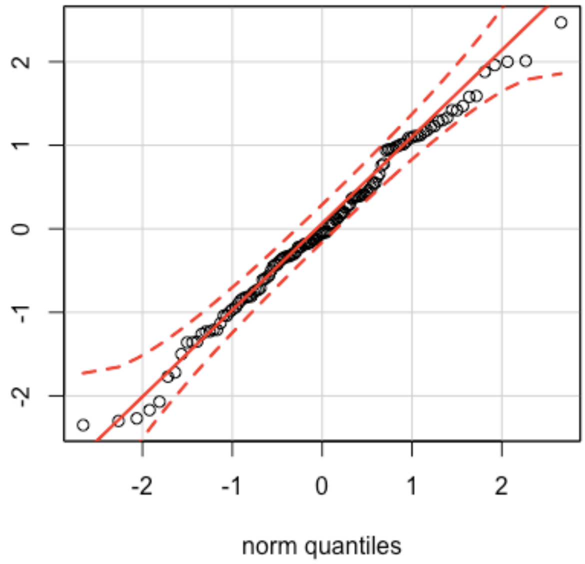

In addition to the graph's inspection, also the Kolmogorov-Smirnoff test (D = 0.06, p =0.53) and the Shapiro-Wilk Test (W = 0.99, p = 0.58) confirmed that data were normally distributed.

b) For what concerns the section: **"Longitudinal effect of intensive music training"**

Before running Generalized Linear Models (GLMMs) in which each factor extracted by the PCA was used as dependent variable, the ID of participants as random intercept, Group and Time as fixed effect, the empirical distributions of the dependent variables were evaluated on the basis of boxplots' inspection and of theoretical model fitting. Here is worth noting that we preferred to remove the outliers for normalizing the distribution instead of using the Gamma family distribution, because the algorithm did not converge when trying to test the group-by-time interaction effects with the Gamma distribution.

Factor 1: General Cognitive Abilities

1.1 empirical data distribution and box-plots

n	*Mean*	*sd*	*Min.*	*Max.*	*Skewness*	*Kurtosis*
255	-0.005	1.008	-1.930	4.640	0.442	-0.304

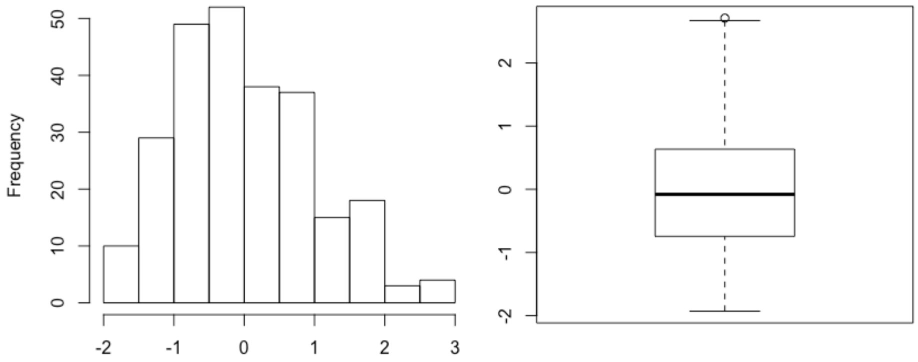

1.2 theoretical model fitting

Normal Distribution:

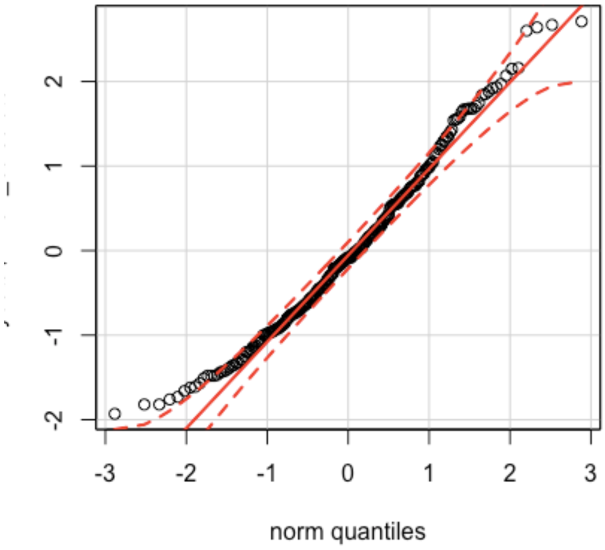

Factor 2: Speed of Linguistic Elaboration

2.1 empirical data distribution and box-plots

n	*Mean*	*sd*	*Min.*	*Max.*	*Skewness*	*Kurtosis*
255	-0.004	0.786	-1.580	6.380	1.549	3.241

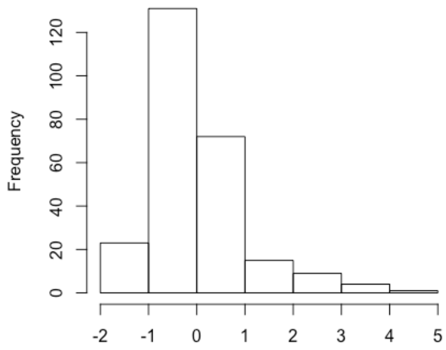

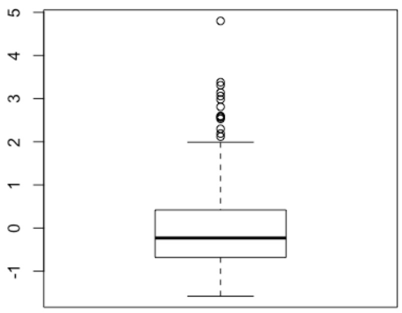

On the basis of boxplot, 14 outliers (out of 255 measures) were identified. To better describe these outliers we also reported the Cook distance plot:

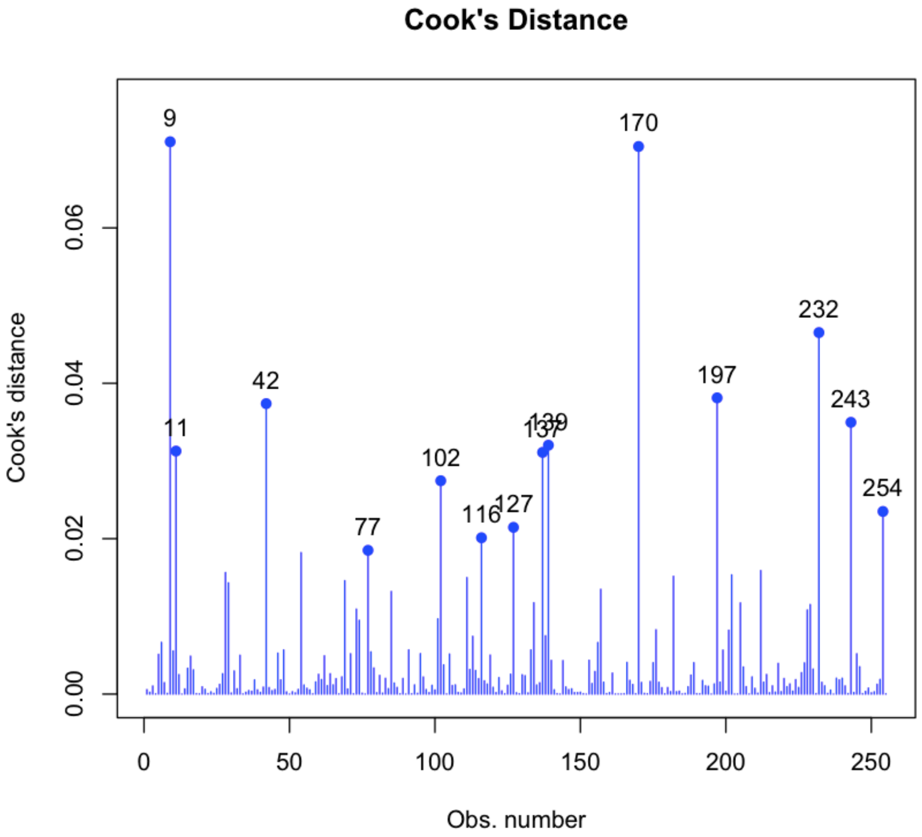

2.1 empirical data distribution and box-plots after outliers' removal

n	*Mean*	*sd*	*Min.*	*Max.*	*Skewness*	*Kurtosis*
241	-0.171	0.703	-1.580	1.990	0.562	-0.096

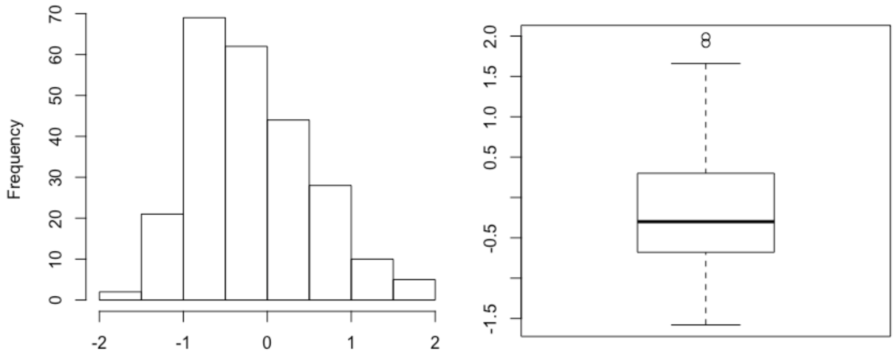

2.2 theoretical model fitting

Normal Distribution (after removing 14 outliers):

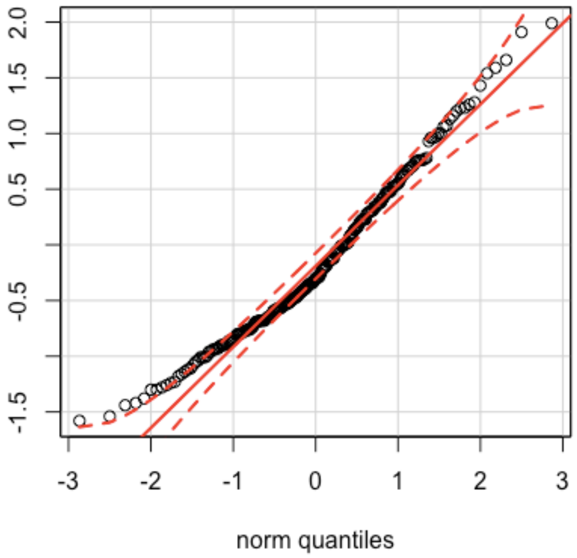

Factor 3: Accuracy in Reading and Memory tests

3.1 empirical data distribution and box-plots

n	*Mean*	*sd*	*Min.*	*Max.*	*Skewness*	*Kurtosis*
255	0.004	0.904	-1.800	5.760	0.947	1.620

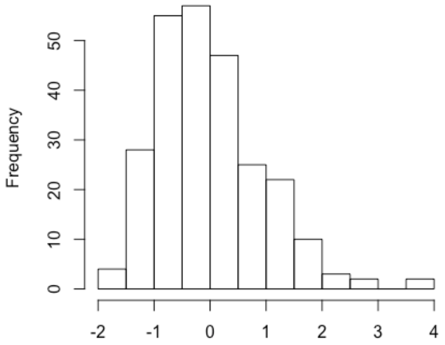

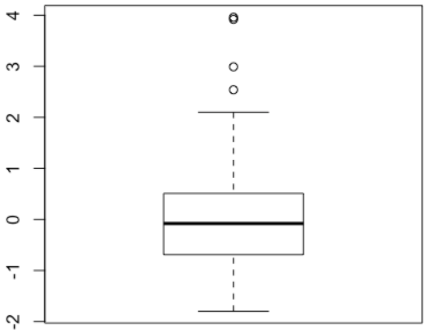

On the basis of boxplot, 7 outliers (out of 255 measures) were identified. To better describe these outliers we also reported the Cook distance plot:

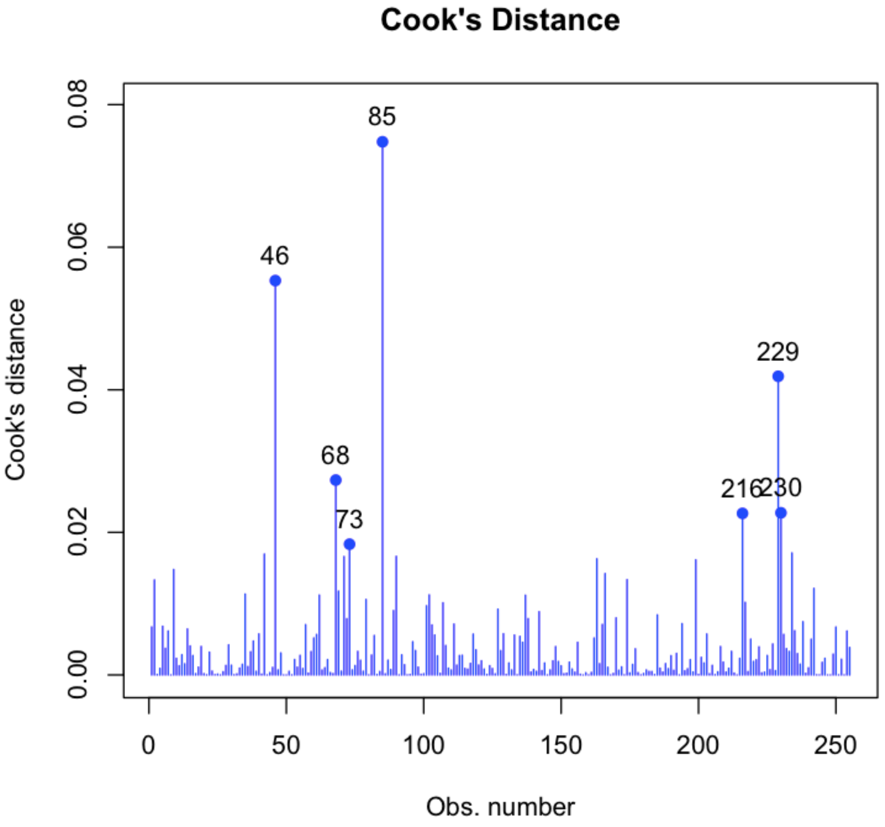

2.1 empirical data distribution and box-plots after outliers' removal

n	*Mean*	*sd*	*Min.*	*Max.*	*Skewness*	*Kurtosis*
248	-0.075	0.813	-1.800	1.890	0.355	-0.520

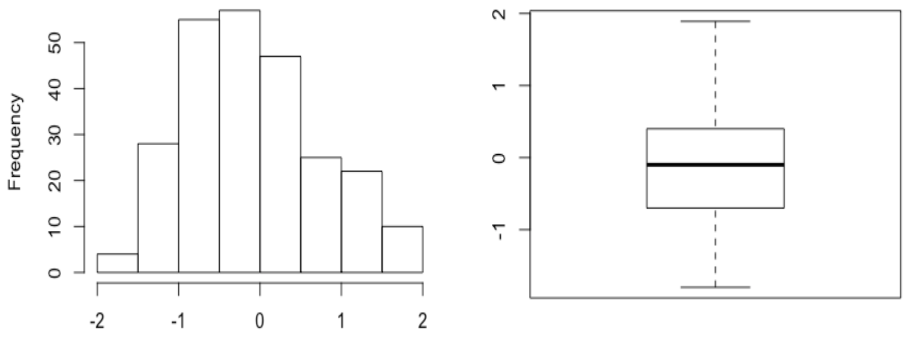

3.2 theoretical model fitting

Normal Distribution (after removing 7 outliers):

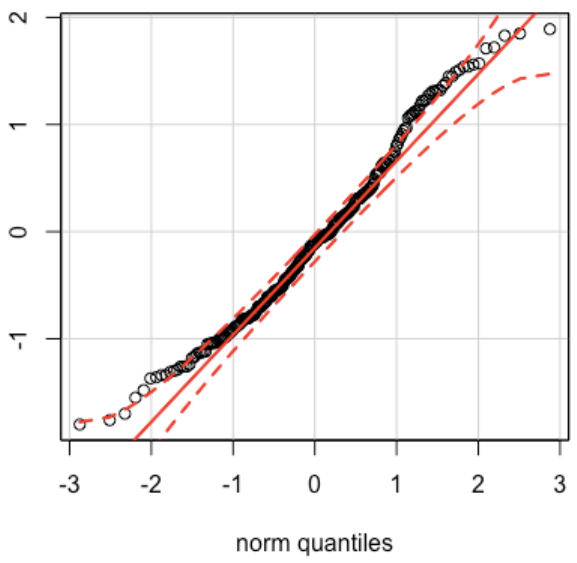

Factor 4: Visuo-spatial and numerical skills

4.1 empirical data distribution and box-plots

n	*Mean*	*sd*	*Min.*	*Max.*	*Skewness*	*Kurtosis*
255	0.004	0.964	-3.130	6.400	-0.124	0.413

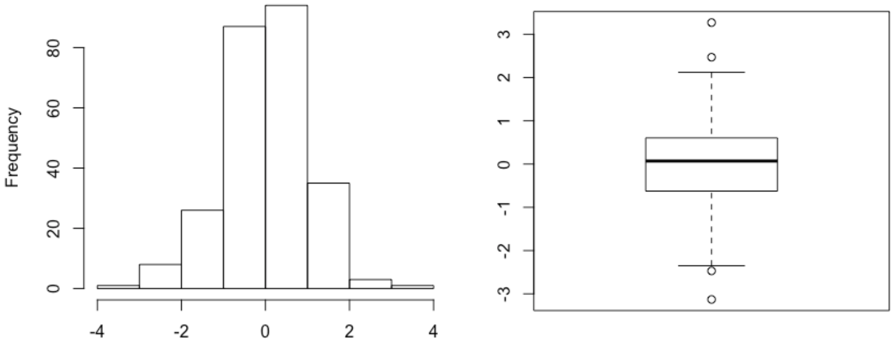

3.2 theoretical model fitting

Normal Distribution:

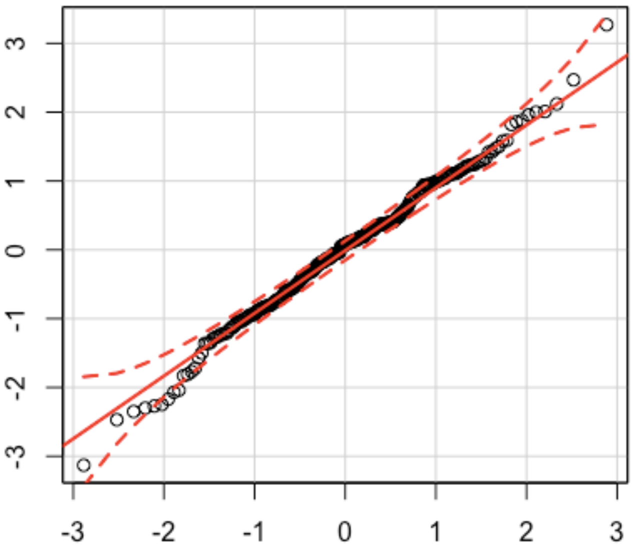

Supplement: Supplementary file 1 [file Data_Sheet_1.pdf]
